# Serabelisib, a Selective PI3Kα Inhibitor, for Slow-Flow Vascular Malformations in a Phase 3 Multicenter Single-Arm Study with Natural History Comparison

**DOI:** 10.3390/jcm15218624

**Published:** 2026-11-05

**Authors:** Noriko Aramaki-Hattori, Satoru Sasaki, Naoki Murao, Taiki Nozaki, Akihiro Fujino, Ayato Hayashi, Michio Ozeki, Saori Endo, Shimpei Akiyama, Tadashi Nomura, Shunsuke Sakakibara, Yoshihisa Kawakami, Ai Morita, Junko Ochi, Motoki Akamatsu, Sadanori Akita

**Affiliations:** 1 Department of Plastic and Reconstructive Surgery, Keio University School of Medicine, Tokyo 160-8582, Japan; 2 Department of Plastic Surgery, Tonan Hospital, Sapporo 060-0004, Japan; 3 Department of Radiology, Keio University School of Medicine, Tokyo 160-8582, Japan; 4 Department of Pediatric Surgery, Keio University School of Medicine, Tokyo 160-8582, Japan; 5 Department of Plastic and Reconstructive Surgery, Yokohama City University School of Medicine, Yokohama 236-0004, Japan; 6 Department of Pediatrics, Gifu University Graduate School of Medicine, Gifu 501-1194, Japan; 7 Department of Radiology, Kyoto Prefectural University of Medicine, Kyoto 602-8566, Japan; 8 Department of Plastic Surgery, Kobe University Graduate School of Medicine, Kobe 650-0017, Japan; 9 Department of Plastic Surgery, National Hospital Organization Kobe Medical Center, Kobe 654-0155, Japan; 10 Department of Plastic Surgery, Fukuoka Children’s Hospital, Fukuoka 813-0017, Japan; 11 Department of Plastic and Reconstructive Surgery, Osaka Rosai Hospital, Sakai 591-8025, Japan; 12 Department of Radiology, University of Iowa Hospitals and Clinics, Iowa City, IA 52242, USA; 13 Kaken Pharmaceutical Co., Ltd., Tokyo 113-8650, Japan; 14 Department of Bioregulation and Pharmacological Medicine, Fukushima Medical University, Fukushima 960-1295, Japan; 15 Department of Plastic Surgery, Tamaki Aozora Hospital, Tokushima 779-3125, Japan

**Keywords:** serabelisib, phosphoinositide 3-kinase α inhibitor, venous malformation, lymphatic malformation, Klippel–Trenaunay syndrome

## Abstract

**Background/Objectives**: Slow-flow vascular malformations, including venous malformation (VM), lymphatic malformation (LM), and Klippel–Trenaunay syndrome (KTS), are rare, symptomatic disorders with limited treatment options. This study evaluated the efficacy and safety of serabelisib, an orally available selective phosphoinositide 3-kinase α inhibitor, in these patients. **Methods**: This phase 3, multicenter, single-arm, confirmatory study was conducted in Japan (September 2023–June 2025). Patients aged ≥2 years received oral serabelisib once daily (100 mg for body weight ≥ 40 kg; 60 mg/m^2^ for <40 kg) for up to 52 weeks. The primary endpoint was the response rate at the final evaluation (≥24 weeks), defined as the proportion of patients achieving a ≥20% reduction from baseline in target lesion volume on blinded, centrally reviewed magnetic resonance imaging. The response rate was compared with a natural history-based null rate of 6.7% (exact binomial test). Trial registration: jRCT2041230064. **Results**: Thirty-six patients received serabelisib. The response rate was 30.6% (11/36; 95% confidence interval, 16.3–48.1%; *p* < 0.001) and remained significant in post hoc sensitivity analyses for null rates of up to 16%. Response rates were 19.0% (VM), 33.3% (LM), and 50.0% (KTS). Quality of life did not improve consistently. Adverse drug reactions (ADRs) occurred in 52.8% of patients (grade 3 or 4, 22.2%), most commonly hyperinsulinemia (13.9%), nausea (11.1%), and liver function test abnormal (11.1%); most hepatic ADRs (13 of 15 events) resolved after dose interruption or discontinuation. One death (tubulointerstitial nephritis) was judged unrelated to treatment. **Conclusions**: Serabelisib produced radiological responses exceeding a natural history benchmark in refractory VM, LM, and KTS, with hepatic toxicity requiring monitoring. Larger controlled studies with longer follow-up are needed to confirm its clinical benefit and long-term safety.

## 1. Introduction

Venous malformation (VM), lymphatic malformation (LM), and Klippel–Trenaunay syndrome (KTS), which involves combined vessels, are slow-flow vascular malformations [1] that present with clinical manifestations ranging from mild to life-threatening, including pain, ulceration, abnormal limb growth, functional impairment, and esthetic concerns [2,3,4]. These diseases are diagnosed primarily on the basis of clinical findings and imaging studies such as magnetic resonance imaging (MRI) and ultrasound, with pathologic examination if needed [2]. VM, LM, and KTS are rare, with prevalences of 1 to 2 per 10,000, 1 per 6000 to 16,000, and 1 per 100,000, respectively [5,6,7].

Current management of VM, LM, and KTS consists of compression therapy with elastic garments and invasive treatments, including surgical resection, sclerotherapy/embolization, and neodymium-doped yttrium aluminum garnet (Nd:YAG) or semiconductor diode laser therapy [2,8]. However, some patients (e.g., those with glomuvenous malformation) experience worsening pain with compression therapy, and there are lesion sites to which this therapy cannot be applied. Other patients are not candidates for invasive therapy or show an insufficient response to it [2,8]. Recent advances in genetics have revealed that certain slow-flow vascular malformations share mutations with cancers, specifically in the phosphoinositide 3-kinase (PI3K)/protein kinase B (AKT)/mammalian target of rapamycin (mTOR) proliferative pathway [9].

Sirolimus, an mTOR inhibitor traditionally used as an immunosuppressive agent that also has antiangiogenic and antineoplastic properties [10], is effective in the treatment of slow-flow vascular malformations and is used clinically [11,12]. Sirolimus has expanded the treatment options for these diseases; however, it also poses safety concerns, such as interstitial pneumonia and infection, owing to its immunosuppressive effects [10,11,12]. In addition, somatic gain-of-function mutations in *PIK3CA*, the gene encoding phosphoinositide 3-kinase α (PI3Kα), have been identified in patients with VM, LM, and KTS [13,14,15,16], suggesting that a selective PI3Kα inhibitor could be an effective and safe treatment option. In the United States, the PI3Kα inhibitor alpelisib received accelerated approval in 2022 for a severe *PIK3CA*-related overgrowth spectrum [17]. Although alpelisib is a selective PI3Kα inhibitor, it does not completely spare PI3K isoform δ (PI3Kδ) inhibition [18], and concerns remain regarding potential adverse effects such as rash, which are known to be associated with PI3Kδ inhibition [19]. A more highly selective PI3Kα inhibitor could therefore theoretically produce fewer immunosuppressive effects, although this hypothesis has not been tested in comparative studies.

Serabelisib is an orally available, selective PI3Kα inhibitor originally developed for advanced solid tumors [20]. Previous phase 1 and phase 2 studies have suggested its potential utility in the treatment of symptomatic and refractory VM, LM, and KTS [21,22]. Because these are rare diseases, it was difficult to establish a concurrent placebo control group in a pivotal clinical study and validate the efficacy and safety of serabelisib in these populations. We therefore first conducted a prospective observational study to characterize the natural history of these vascular malformations [22,23]. Using the observed natural history data as the null hypothesis response rate, we then evaluated the benefit of serabelisib in this single-arm phase 3 study with natural history comparison.

## 2. Materials and Methods

### 2.1. Study Design

This phase 3, multicenter, single-arm, confirmatory study was conducted at six medical institutions in Japan from 14 September 2023 to 20 June 2025. The study evaluated the efficacy, safety, and pharmacokinetics (PK) of serabelisib administered to Japanese patients with VM, LM, or KTS for at least 24 weeks and up to 52 weeks. The study was registered in the Japan Registry of Clinical Trials (jRCT2041230064) before its initiation.

### 2.2. Patients

Eligible patients were Japanese individuals aged 2 years or older at the time of consent who had been diagnosed with symptomatic and refractory VM, LM, or KTS according to the Japanese diagnostic criteria for vascular malformations [24,25,26] and the International Society for the Study of Vascular Anomalies (ISSVA) classification [1]. Patients were enrolled if they had at least one lesion measurable by MRI. Exclusion criteria were diabetes mellitus (type 1 or type 2); disorders causing abnormal glucose metabolism (e.g., glycogen storage disease, galactosemia, primary lactose intolerance); hepatic or renal impairment; ischemic heart disease, arrhythmia, or heart failure (New York Heart Association class III or IV) deemed inadequately controlled; and gastrointestinal disorders affecting drug absorption.

### 2.3. Treatment

Patients received serabelisib dry syrup orally once daily after breakfast for up to 52 weeks in an unblinded manner. The dose was 100 mg/day for patients weighing 40.0 kg or more. For patients weighing less than 40.0 kg, the daily dose was calculated from the body surface area using the Du Bois formula:

Body surface area (m^2^) = weight (kg)^0.425^ × height (cm)^0.725^ × 0.007184

Dose (mg) = 60 mg/m^2^ × body surface area (m^2^)

When adverse events (AEs) occurred, dose reduction to half the assigned dose or treatment discontinuation was permitted on the basis of the patient’s condition.

### 2.4. Assessment

#### 2.4.1. Efficacy

The primary endpoint was the response rate, defined as the proportion of patients who achieved a ≥20% reduction from baseline in target lesion volume determined by MRI at the final evaluation point after at least 24 weeks and up to 52 weeks of serabelisib administration. The final evaluation point was defined as the last MRI assessment performed after at least 24 weeks of treatment, that is, week 52 for patients who completed the study and week 24 for patients who discontinued treatment between weeks 24 and 52. Patients without an evaluable MRI assessment at or after week 24 were classified as non-responders. Patients without an evaluable baseline target lesion volume, for whom the percent change could not be calculated, were also classified as non-responders. Complete response, partial response, stable disease, and progressive disease were defined as disappearance of the target lesion, a ≥20% reduction without disappearance, a <20% reduction or <20% increase, and a ≥20% increase from the baseline volume, respectively. The ≥20% threshold was adopted because it exceeds the expected variability in volumetric measurements and is consistent with the response criteria used in the natural history study from which the null hypothesis rate was derived [22,23] and in a previous trial of sirolimus for lymphatic anomalies [27]; however, it has not been validated as a threshold of clinically meaningful improvement. To ensure the objectivity of these assessments, target lesion volumes were measured by an independent central Image Evaluation Committee. MRI was performed at baseline and at weeks 24 and 52 using a standardized imaging protocol common to all study sites (1.5-T or 3-T scanners) with the requirement that each patient be scanned using the same device and identical parameters throughout the study. Target lesion volumes were measured on axial fat-suppressed T2-weighted or short tau inversion recovery (STIR) images with a slice thickness ranging from 3 to 5 mm (up to 8 mm for large lesions), which was kept constant for each patient across all time points, without an interslice gap. The Chair of the Image Evaluation Committee and the reviewers jointly defined the measurement range for each patient on the basis of bony and other anatomical landmarks, ensuring that the same anatomical range was evaluated at each time point. Within this range, the lesion contour was delineated on each slice with the semi-automated region-growing tool of OsiriX MD software (version 14, Pixmeo SARL, Bernex, Switzerland) and refined manually with the brush and pencil tools to exclude non-lesional structures that were often captured inadvertently by the semi-automated tool, such as normal blood vessels, and to correct the contour when the lesion boundary could not be captured automatically (e.g., infiltrative lesions or lesions adjacent to structures with similar signal intensity). The lesion volume was calculated as the sum of the delineated areas multiplied by slice thickness. The measurement procedure is illustrated in supplementary materials. The images of each patient were measured independently by two radiologist reviewers who were blinded to clinical information. The Chair of the Image Evaluation Committee reviewed the results from both reviewers; if a measurement was considered inappropriate, the Chair discussed it with the reviewers and requested re-measurement as necessary. Although the reviewers were aware of the chronological order of the images to allow for comparative assessment, they were blinded to all other clinical data. Each reviewer submitted their measurement records to the sponsor via a secure cloud server. The final target lesion volume for each time point was then calculated by the sponsor as the mean of the values provided by the two reviewers. These volumes were used to determine the response rate.

Secondary endpoints included the response rate at weeks 24 and 52, the change from baseline in target lesion volume, the pain score assessed with a visual analog scale (VAS), quality of life (QOL), and performance status. Pain scores were prespecified to be analyzed separately in patients with a baseline VAS score of ≥10 mm. QOL was assessed with the Pediatric Quality of Life Inventory (PedsQL) 4.0 Generic Core Scales (ages 2–4 years, parent proxy report; ages 5–18 years, patient report and parent proxy report; ages 18–25 years, patient report) [28,29,30,31], the 36-Item Short Form Health Survey version 2 (SF-36v2; age 26 years and older) [32,33,34], the EuroQol 5-Dimension Youth questionnaire (EQ-5D-Y; ages 4–7 years, parent proxy report; ages 8–15 years, patient report) [35], and the EQ-5D-5L (age 16 years and older, patient report) [36]. Performance status was evaluated with either the Lansky play-performance scale (younger than 17 years) [37] or the Karnofsky performance scale (17 years and older) [38]. Other assessments included disease severity in the cosmetic and functional domains, blood coagulation parameters, and the response rate in non-target lesions, defined as lesions other than the target lesion.

#### 2.4.2. Safety

Treatment-emergent AEs were defined as AEs that occurred or worsened in severity after the first dose of study treatment. The incidence of AEs, clinical laboratory test results, and vital signs were monitored throughout the study period. AEs were coded by system organ class and preferred term according to the Medical Dictionary for Regulatory Activities (MedDRA) version 28.0, and AE terms in this manuscript are presented as MedDRA preferred terms (PTs). The investigator graded the severity of each AE according to the National Cancer Institute Common Terminology Criteria for Adverse Events (CTCAE version 5.0). The seriousness of each AE was assessed by the investigator according to International Council for Harmonisation (ICH) standards. An adverse drug reaction (ADR) was defined as an AE considered by the investigator to be related to serabelisib treatment. Causality was assessed by the investigator on the basis of the temporal relationship to treatment, the course of the event after dose interruption or discontinuation, the presence of alternative causes, and the known pharmacology of PI3K inhibitors. The AEs designated as safety risks of interest for serabelisib were prespecified in the study protocol and comprised hyperglycemia, increased blood creatine phosphokinase, increased alanine aminotransferase (ALT) increased, and increased aspartate aminotransferase (AST).

#### 2.4.3. Pharmacokinetics

To evaluate the steady-state trough concentration of serabelisib, plasma concentrations of serabelisib were measured before administration at weeks 12, 24, 36, and 52.

### 2.5. Statistical Analysis

In this single-arm study without a control arm, the response rate after serabelisib treatment was compared statistically with a null hypothesis response rate derived from the natural history of the diseases. A previous post hoc analysis of a natural history study of patients with intractable VM and KTS showed a response rate of 6.7% (2 of 30 patients) after a 6-month observation period without any intervention [22,23]. For LM, a retrospective national survey of untreated intractable lymphatic anomalies, reported in the protocol of a sirolimus trial, showed a response rate of 1.7% (1 of 59 patients) over at least 1 year, including 5.3% (1 of 19 patients) among patients with cystic LM [27]. The response rate after serabelisib treatment was therefore compared with a null hypothesis response rate of 6.7%, the higher of these benchmarks, using an exact binomial test. The 95% confidence intervals (CIs) for the response rates were calculated using the Clopper–Pearson exact method. Response rates were also summarized by disease type, body weight (<40 kg and ≥40 kg), and baseline target lesion volume (<200 cm^3^, 200 to <1000 cm^3^, and ≥1000 cm^3^). In a previous phase 2 study in patients with VM, LM, and KTS, serabelisib showed a response rate of 33.3% [22]. Assuming an expected response rate of 30% with serabelisib, a two-sided α of 0.05, and a power of 90%, 29 patients were required when the null hypothesis rate of 6.7% was used as the threshold. Because the expected discontinuation or dropout rate was approximately 10%, the target sample size was set at 32 patients. In the analysis of the response rate for the target lesion, patients with missing data were considered non-responders. For all other analyses, missing data were not imputed. All *p*-values were two-sided and were not adjusted for multiple testing, and *p*-values of less than 0.05 were considered statistically significant. Interobserver agreement for target lesion volume measurements was assessed with Bland–Altman analysis, in which the bias (mean difference between reviewers) and 95% limits of agreement (LoA) were estimated and the bias was tested with a paired *t*-test. Because the pooled analysis included up to three paired measurements per patient, which are not independent [39], the Bland–Altman analysis was also performed separately for each visit (screening and weeks 24 and 52), so that each patient contributed one measurement pair to each analysis. To express the variability relative to lesion size, which ranged widely, the visit-specific analyses were repeated using the percentage difference between the reviewers (difference divided by the mean of the two measurements). The following analyses were performed post hoc: the visit-specific Bland–Altman analyses of absolute and percentage differences; the classification of responders into those who responded at both week 24 and 52 (early responders) and those who responded at week 52 only (late responders); and a descriptive comparison of changes in pain scores, QOL scores, and performance status between responders and non-responders. Plasma concentrations below the limit of quantification (BLQ) were regarded as zero in the summary statistics. Statistical analyses were performed with SAS version 9.4 (SAS Institute Inc., Cary, NC, USA).

Because the historical response rate is itself an estimate based on a small cohort, the following post hoc sensitivity analyses were performed: (i) exact binomial tests against alternative null hypothesis rates (1.7%, 10%, 15%, 20%, and 22.1%, the upper limit of the 95% CI of the natural history response rate) and identification of the highest null hypothesis rate at which the result remained significant at a one-sided α of 0.025; (ii) Fisher’s exact test comparing the proportion of responders with that in the natural history cohort (2 of 30 patients), which treats the historical rate as an estimate rather than a fixed value; (iii) a Bayesian analysis with Jeffreys priors (Beta [0.5, 0.5]) for both response rates, estimating the posterior probability that the response rate with serabelisib exceeded the natural history rate; and (iv) analyses restricted to patients with VM or KTS, the diseases included in the natural history study, and to patients who completed 52 weeks of treatment.

### 2.6. Use of Generative Artificial Intelligence

During the preparation of this manuscript and its revision, the authors used Claude Opus 5.5 (Anthropic, San Francisco, CA, USA) for English-language editing, formatting according to the journal’s instructions, checking the consistency of the text, tables, and references, drafting revised wording of selected passages (including the Abstract Section, Discussion Section, and responses to the reviewers), and computing the post hoc sensitivity analyses from aggregate results, which were verified by the authors. Generative AI was not used for study design, data collection, primary statistical analyses, or the generation of figures. The authors reviewed and edited all AI-assisted content and take full responsibility for the content of this publication.

## 3. Results

### 3.1. Patient Disposition

**Figure 1:**
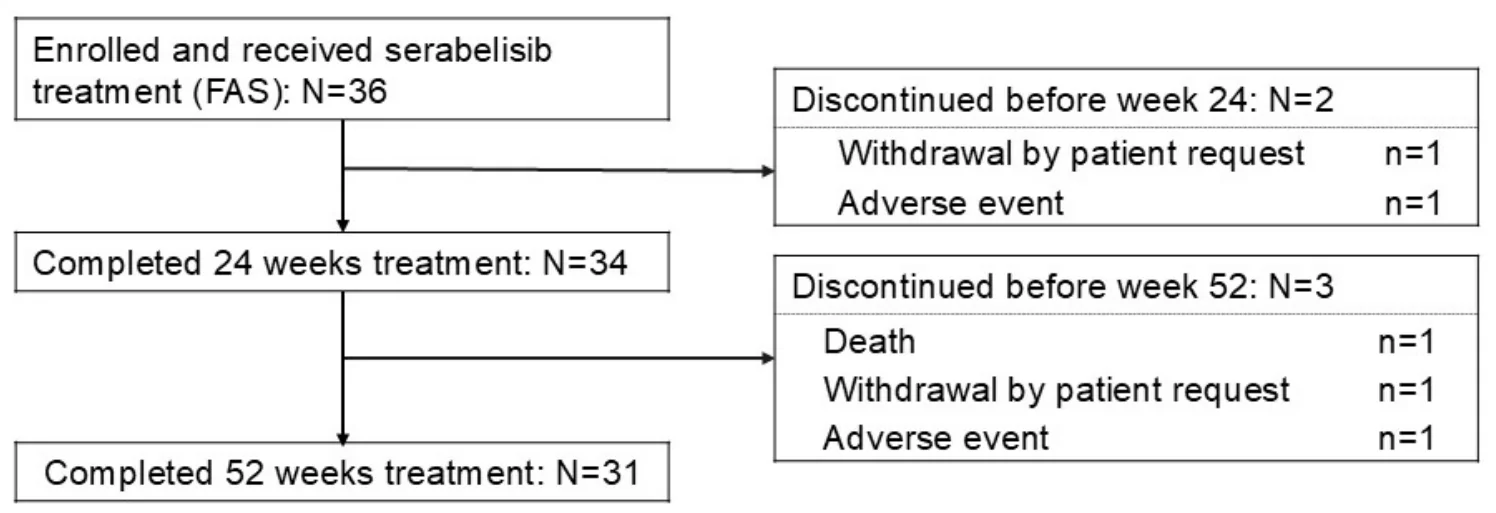
Flow diagram of patient disposition. FAS, full analysis set.

Thirty-six patients were enrolled in the study and received serabelisib (full analysis set [FAS]). Of these, 34 (94.4%) and 31 (86.1%) completed 24 and 52 weeks of serabelisib treatment, respectively (Figure 1). The final evaluation point was week 52 for the 31 patients who completed treatment and week 24 for the 3 patients who discontinued treatment between weeks 24 and 52; the 2 patients who discontinued before week 24 had no evaluable assessment at or after week 24 and were classified as non-responders. Thus, the final evaluation point differed from the week 52 assessment only in the three patients evaluated at week 24, none of whom met the response criterion; consequently, the response rate at the final evaluation point was identical to that at week 52.

### 3.2. Patient Characteristics

In the FAS, 55.6% of patients were male and 44.4% were female. VM, LM, and KTS accounted for 58.3%, 8.3%, and 33.3% of patients, respectively. The mean age was 21.6 ± 18.0 years, and 55.6% of patients were younger than 18 years (Table 1). Target lesions were located in the lower limb in 12, the upper limb in 8, the head and neck in 12, and the trunk in 4 patients. The median baseline target lesion volume was 241.2 cm^3^ (range, 3.1–2728.8 cm^3^; *n* = 35) (Table S4).

### 3.3. Efficacy

**Figure 2:**
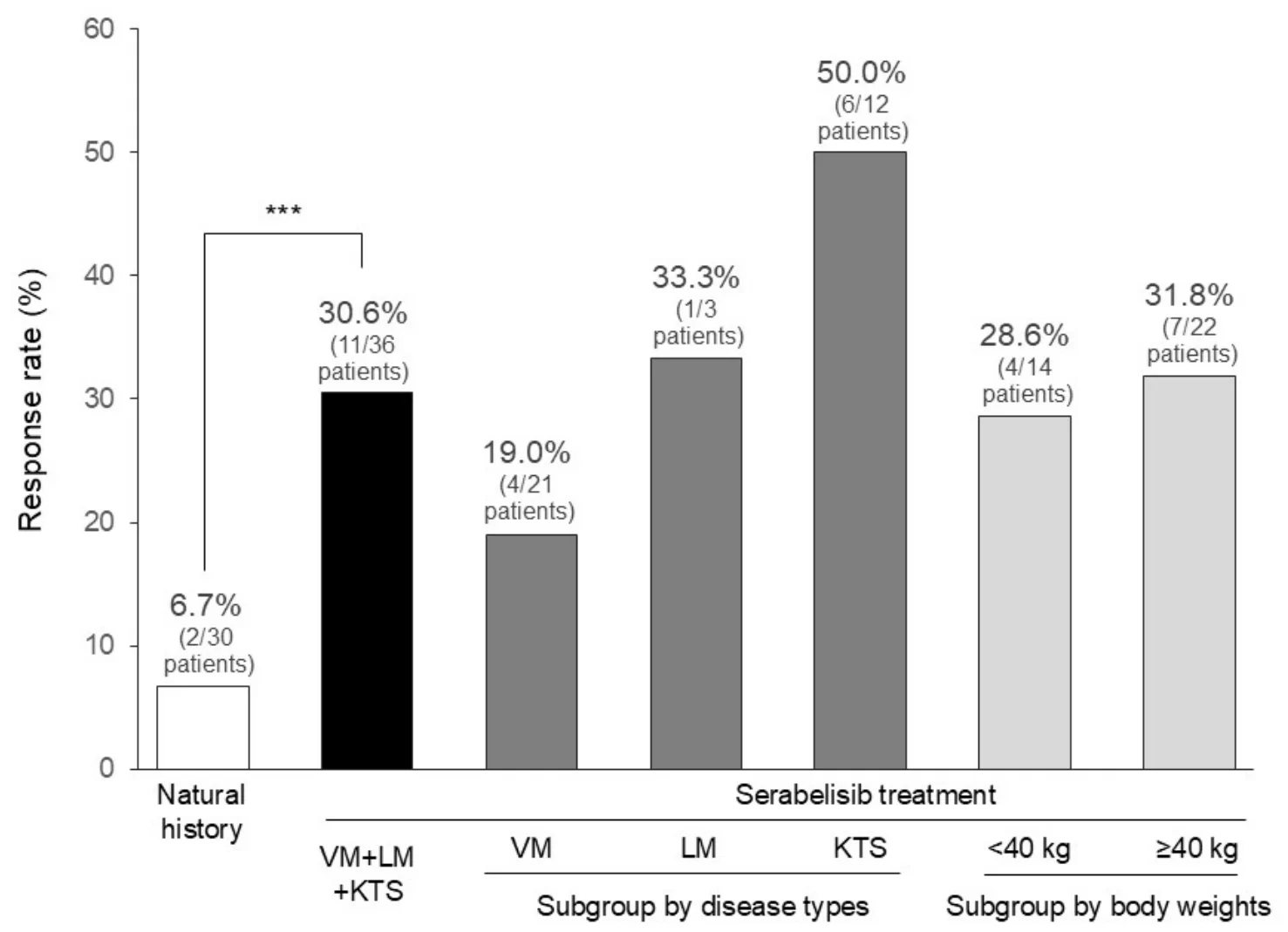
Response rate at the final evaluation point. Response rate was defined as the proportion of patients with a complete or partial response. *** *p* < 0.001 vs. the null hypothesis rate of 6.7% derived from natural history data (exact binomial test). KTS, Klippel–Trenaunay syndrome; LM, lymphatic malformation; VM, venous malformation.

In the FAS, the response rate at the final evaluation point (the primary endpoint) was 30.6% (11 of 36 patients; 95% CI, 16.3–48.1%), which was significantly higher than the prespecified null hypothesis rate of 6.7% (*p* < 0.001, exact binomial test). Numerically, response rates were 19.0% (4 of 21; 95% CI, 5.4–41.9%) for VM, 33.3% (1 of 3; 95% CI, 0.8–90.6%) for LM, and 50.0% (6 of 12; 95% CI, 21.1–78.9%) for KTS; these subgroup findings are descriptive and do not allow for comparisons between disease types because of the small number of patients, particularly in the LM subgroup. The response rate was similar in the subgroup weighing less than 40 kg (28.6%, 4 of 14) and that weighing 40 kg or more (31.8%, 7 of 22) (Figure 2). The change from baseline in target lesion volume in each patient is shown in Figure S1, and absolute target lesion volumes at baseline and at weeks 24 and 52 are shown in Table S4. The median target lesion volume was 241.2 cm^3^ (range, 3.1–2728.8 cm^3^; *n* = 35) at baseline and 216.5 cm^3^ (range, 4.4–2019.7 cm^3^; *n* = 31) at week 52. By baseline target lesion volume, response rates were 26.7% (4 of 15) for volumes < 200 cm^3^, 31.6% (6 of 19) for 200 to <1000 cm^3^, and 100% (1 of 1) for ≥1000 cm^3^; these estimates are descriptive because of the small subgroup sizes. In the Bland–Altman analysis of 101 paired measurements, the mean volumes measured by the two reviewers were 351.2 cm^3^ and 352.1 cm^3^, respectively. The bias between the reviewers was 0.90 cm^3^ (95% CI, 0.01–1.79 cm^3^; *p* = 0.047), and the 95% LoA ranged from −7.93 to 9.74 cm^3^. Although statistically significant, the bias was small relative to the median target lesion volume (241.2 cm^3^), indicating good interobserver agreement. In the visit-specific analyses, the 95% LoA ranged from −7.07 to 9.55 cm^3^ at screening, from −4.73 to 5.71 cm^3^ at week 24, and from −11.22 to 13.15 cm^3^ at week 52. The bias in percentage differences was 0.33% (95% LoA, −1.38% to 2.04%) at screening, 0.24% (95% LoA, −2.49% to 2.97%) at week 24, and 0.01% (95% LoA, −2.53% to 2.54%) at week 52; thus, the interobserver variability was considerably smaller than the 20% response threshold (Figure S4). The Chair of the Image Evaluation Committee reviewed all segmentations and requested re-measurement in 7 of 36 patients (19.4%); the corrected measurements were approved by the Chair and used in the final analysis.

Among the secondary endpoints, the response rate was 22.2% (8 of 36 patients; 95% CI, 10.1–39.2%) at week 24 and 30.6% (11 of 36 patients) at week 52, both of which were significantly higher than the prespecified null hypothesis rate (*p* = 0.004 and *p* < 0.001, respectively). The mean percent changes from baseline in target lesion volume were −5.08 ± 19.35% at week 24 (*n* = 32) and −6.83 ± 24.52% at week 52 (*n* = 30); percent changes could not be calculated for one patient without an evaluable baseline volume, for one patient whose MRI at week 24 was missing because of a protocol deviation, and for patients who had withdrawn from the study (two at week 24 and five at week 52). Individual changes varied considerably among patients (Figure S1); the mean reduction in target lesion volume was modest, whereas the proportion of responders increased from week 24 to week 52. Of the eight patients who responded at week 24, seven maintained the response at week 52 (early responders) and one no longer met the response criterion at week 52, whereas four patients responded only at week 52 (late responders).

In post hoc sensitivity analyses (Table S3), the response rate remained significantly higher than the null hypothesis rate when the LM-based benchmark of 1.7% or alternative null hypothesis rates of 10% and 15% were assumed (one-sided *p* < 0.001, <0.001, and =0.014, respectively), and the result remained significant at a one-sided α of 0.025 for any null hypothesis rate of up to 16%. When the upper limit of the 95% CI of the natural history response rate (22.1%) was used, the difference was not significant (one-sided *p* = 0.15). When the natural history rate was treated as an estimate, the response rate was significantly higher than that in the natural history cohort (30.6% vs. 6.7%; Fisher’s exact test, two-sided *p* = 0.027), and the Bayesian posterior probability that the response rate with serabelisib exceeded the natural history rate was 99.4%. The results were consistent in patients with VM or KTS (10 of 33 [30.3%]; Fisher’s exact test vs. the natural history cohort, two-sided *p* = 0.024) and in patients who completed 52 weeks of treatment (11 of 31 [35.5%]; 95% CI, 19.2–54.6%).

**Figure 3:**
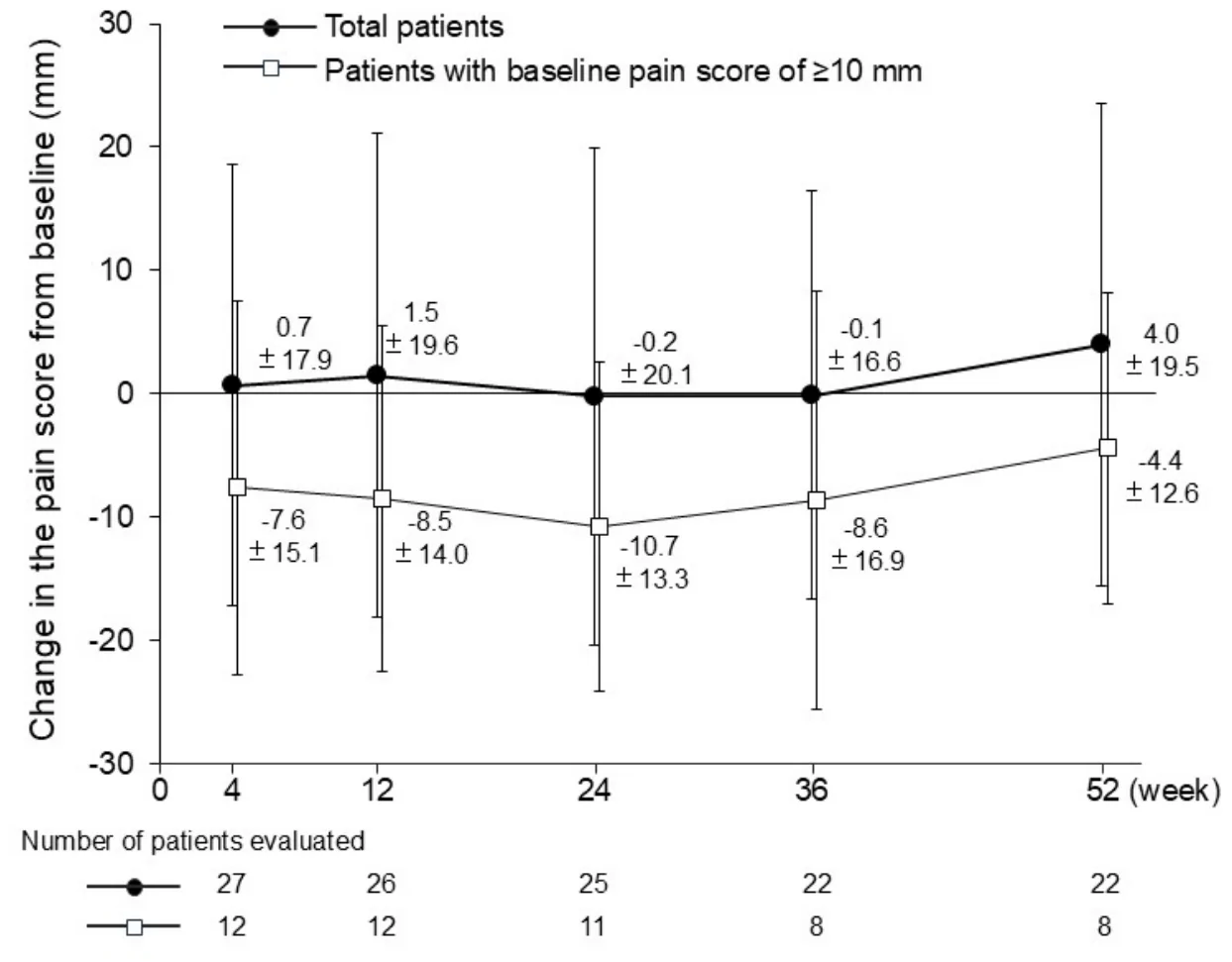
Changes in the pain scores through 52 weeks. Data are expressed as mean ± standard deviation.

The pain score assessed with the VAS (0–100 mm) was evaluated in 27 patients who were capable of self-reporting; the remaining 9 pediatric patients were excluded from this assessment. Among these 27 patients, the change was difficult to interpret because the baseline score was low (mean ± standard deviation [SD], 18.6 ± 24.5 mm). A large proportion of patients (15 of 27) had a baseline VAS score of less than 10 mm. Therefore, pain scores were analyzed separately in patients with a baseline VAS score of ≥10 mm; in this subgroup (*n* = 12), the scores tended to decrease consistently after serabelisib treatment (Figure 3). No notable changes were observed in QOL after serabelisib treatment (Table S1). The PedsQL total scores changed by +3.41 points (parent proxy report, *n* = 18) and +3.55 points (patient report, *n* = 15) from baseline to week 52, which did not reach the minimal clinically important differences of 4.5 and 4.4 points, respectively [40]. The SF-36v2 mental and role/social component summary scores (*n* = 7) and the EQ-5D-5L utility score (*n* = 13) decreased numerically, whereas the EQ-5D-Y scores were stable or increased slightly in small numbers of evaluable patients (*n* = 4–7). On the Lansky play-performance scale (younger than 17 years; *n* = 18), 7 patients showed improved scores from baseline after serabelisib treatment, 8 showed no change, and 3 showed worsening. The Karnofsky performance scale (17 years and older; *n* = 13) improved in 4 patients, was unchanged in 9 patients, and worsened in no patients. In a post hoc descriptive comparison by response status, radiological responders did not show consistently greater improvements than non-responders: the VAS pain score changed by +8.25 ± 22.86 mm in responders (*n* = 8) and by +1.64 ± 17.76 mm in non-responders (*n* = 14), and the PedsQL patient-reported total score changed by −2.45 ± 4.97 points (*n* = 4) and +5.73 ± 7.06 points (*n* = 11), respectively, whereas the PedsQL parent proxy-reported total score changed by +5.34 ± 10.83 points (*n* = 5) and +2.66 ± 9.94 points (*n* = 13), respectively; changes in the SF-36v2, EQ-5D, and performance status scores showed no consistent pattern between the groups (Table S6).

Disease severity in the cosmetic and functional domains showed no notable change after serabelisib treatment, and there were no notable changes in blood coagulation parameters. The response rates in non-target lesions were 30.0% (3 of 10 patients) at week 24 and 40.0% (4 of 10 patients) at week 52.

**Figure 4:**
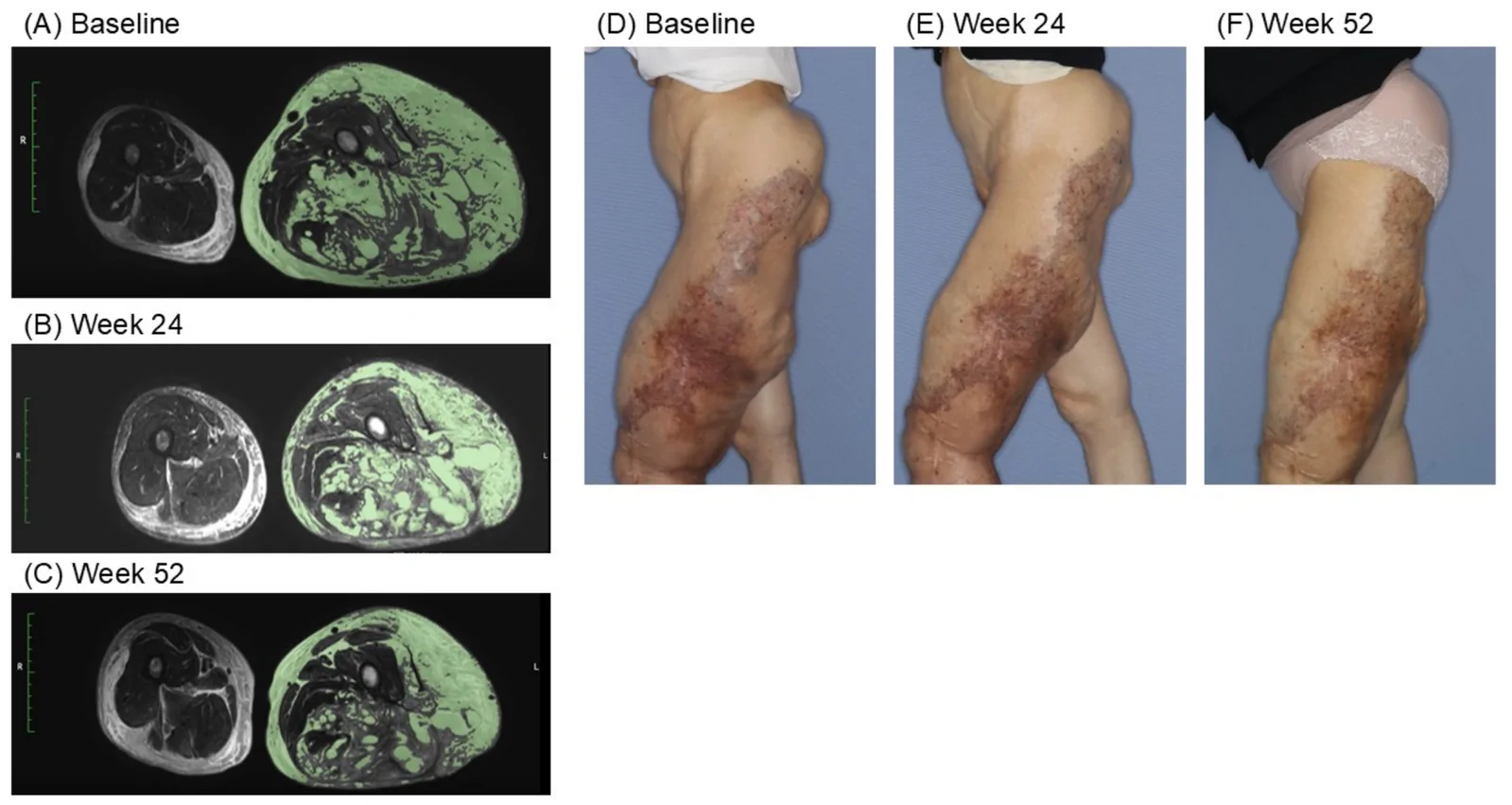
Representative images of the target lesion before and after serabelisib treatment. The magnetic resonance imaging (MRI)-based volumetric analyses of the target lesion (affected left limb) are shown in panels (**A**–**C**) (baseline, week 24, and week 52, respectively), with the lesion highlighted in green. The clinical photographs of the same lesion are shown in panels (**D**–**F**) (baseline, week 24, and week 52, respectively).

As representative images of the efficacy of serabelisib in this study, MRI images and clinical photographs of the target lesion in one patient before and after treatment are shown in Figure 4A–F, demonstrating marked improvement in skin discoloration and swelling after treatment.

### 3.4. Safety

Among the 36 patients, the incidences of AEs and ADRs were 100.0% and 52.8%, respectively. The ADRs occurring at an incidence of 10% or higher were hyperinsulinemia (13.9%), nausea (11.1%), and an abnormal liver function test (11.1%) (Table 2).

Hyperinsulinemia and nausea were mostly grade 1 and did not require dose reduction, dose interruption, or discontinuation of serabelisib treatment. Abnormal liver function tests occurred in four patients (grades 1–3); no dose change was required in one patient, dose interruption was required in two patients, and discontinuation was required in one patient. The two patients who underwent dose interruption restarted serabelisib at 50 mg and subsequently increased the dose to 75 mg, and the event resolved after discontinuation in the patient who discontinued treatment. One 49-year-old female patient died of tubulointerstitial nephritis on day 237 of the study. In this patient, serum creatinine remained within the normal range throughout the treatment period, and there were no symptoms or clinical signs suggestive of renal dysfunction before the patient was found dead at home. Because the cause of this sudden death was unknown, a forensic autopsy was performed and identified tubulointerstitial nephritis as the cause of death. The renal findings were relatively acute, without the interstitial fibrosis that is characteristic of chronic drug-induced kidney injury. The last serum creatinine measurement before death, on day 224, was 0.84 mg/dL. Concomitant medications at the time of death were loxoprofen sodium; heparinoid; a combination cream containing heparinoid, adrenal extract, and salicylic acid; eperisone hydrochloride; sodium ferrous citrate; and fluocinonide. None of these medications had been newly started. Loxoprofen sodium is a nonsteroidal anti-inflammatory drug, a class recognized as a cause of tubulointerstitial nephritis, and therefore represents a possible alternative cause. The autopsy showed scattered hemorrhagic foci with lymphocytic infiltration, tubular injury, and a small number of Langhans giant cells, and no evidence of infection, autoimmune disease, or urinary tract obstruction was found. The investigator judged the event to be unrelated to serabelisib on the basis of its late onset (approximately eight months after treatment initiation), the absence of sudden increases or abnormal fluctuations in serabelisib trough plasma concentrations throughout the study, the absence of tubulointerstitial nephritis in long-term nonclinical toxicity studies and elsewhere in the clinical development program, and the absence of a clear safety signal for acute interstitial nephritis. However, because drug-induced tubulointerstitial nephritis can develop after prolonged drug exposure, a causal relationship with serabelisib cannot be completely excluded.

Other serious AEs occurred in five patients—cellulitis in two patients and ligament injury, Kawasaki’s disease, and bronchitis in one patient each—none of which were considered related to treatment. Grade 3 or 4 ADRs occurred in 8 patients (22.2%). Grade 3 ADRs occurred in 6 patients and included abnormal liver function tests, increased liver function test grade, increased hepatic enzyme levels, erythema multiforme, increased blood creatine phosphokinase levels, and neutropenia. Grade 4 ADRs occurred in two patients: drug-induced liver injury and increased liver function test grade. Both events resolved after dose interruption; one of these patients subsequently discontinued treatment permanently because of a grade 3 liver function test abnormality. ADRs leading to permanent discontinuation were eczema and liver function test abnormality in one patient each, both of which resolved after discontinuation. Overall, dose interruption, dose reduction, and permanent discontinuation because of AEs occurred in 11, 7, and 2 patients, respectively. Hepatic ADRs (liver function test abnormality or increased grade, increased hepatic enzyme, ALT, and AST, and drug-induced liver injury) occurred in 10 patients (15 events); the median time to onset was 59 days (range, 23–288). Of these, 13 events resolved (median time to resolution, 66 days; range, 15–271), and of the remaining 2 events, 1 had not resolved at the end of the study and the outcome of the other was unknown because the patient died before follow-up of this event was completed (Table S5). Details of each hepatic event are provided in Table S5. Regarding the four prespecified safety risks, ADRs included hyperglycemia in one patient (2.8%), increased blood creatine phosphokinase in three (8.3%), and ALT and AST elevations in two each (5.6%). For the patient with hyperglycemia, postprandial blood glucose and hemoglobin A1c remained stable through week 52 (Table S2). There were no clinically meaningful changes in vital signs throughout the study period.

### 3.5. Pharmacokinetics

The steady-state trough concentration of serabelisib was evaluated, and the plasma trough concentrations remained comparable between weeks 12 and 52 of administration (Figure S2).

## 4. Discussion

Treatment options are limited for patients with vascular malformations such as VM, LM, and KTS, which usually appear at birth or in early childhood and can have a severe lifelong impact on QOL, leaving an unmet medical need in these rare diseases [2,3,4,41]. Genetic analyses support the involvement of PI3Kα in the etiology of these diseases [13,14,15,16], and a previous phase 2 study provided proof of concept for the development of the selective PI3Kα inhibitor serabelisib for the treatment of VM, LM, and KTS [22].

In the present phase 3 study, the primary endpoint—the response rate based on a ≥20% reduction from baseline in target lesion volume determined by MRI at the final evaluation point—was 30.6% (95% CI, 16.3–48.1%), which was significantly higher than the prespecified null hypothesis rate of 6.7% [22,23]. This result is consistent with the response rates observed in a previous phase 2 study (29.4% and 33.3% with 50 mg and 100 mg, respectively) [22].

Because the null hypothesis rate was derived from an external natural history cohort, the comparability of the two populations is critical for interpreting this result. The natural history study enrolled patients with intractable VM or KTS at Japanese centers, with an age distribution (1–53 years; median, 15.9 years) similar to that of the present study (2–62 years; median, 15.5 years), and lesion volume was assessed by centrally reviewed, semi-automated MRI volumetry with the same ≥ 20% response criterion [22,23]. In that study, lesion volume did not change significantly over 6 months, and no baseline characteristics affected the change in lesion volume [23]. However, the natural history cohort did not include patients with LM, its observation period (6 months) was shorter than the treatment period in the present study (up to 52 weeks), and the disease severity and previous treatments of the two cohorts could not be formally matched. The 6.7% benchmark was based on only two responders among 30 patients (95% CI, 0.8–22.1%), and the direction of bias arising from the differences between the cohorts is uncertain. The longer assessment period in the present study may have allowed for more spontaneous fluctuations in lesion volume, for example because of intralesional thrombosis, hemorrhage, or inflammation, or delayed regression after previous treatments, which could have inflated the response rate; conversely, the enrollment of patients with refractory lesions may have reduced the likelihood of spontaneous regression. In addition, the null hypothesis rate for LM, which was not represented in the natural history cohort, relied on a separate retrospective survey [27]. The post hoc sensitivity analyses that treated the historical rate as an estimate, restricted the population to VM and KTS, or assumed alternative benchmarks of up to 16% support the primary result; that is, the conclusion would change only if the true spontaneous response rate exceeded approximately 16%, more than twice the prespecified rate. However, the difference was not significant when the upper confidence limit of the historical rate (22.1%) was assumed. Therefore, the present findings support the radiological activity of serabelisib, but the magnitude of benefit relative to the natural course of these diseases remains uncertain.

Regarding the secondary endpoints, the proportion of responders increased from 22.2% at week 24 to 30.6% at week 52, suggesting that responses were maintained or developed with continued treatment. However, the mean reduction in target lesion volume was modest (−6.83% at week 52), and individual changes varied considerably, indicating that only a subset of patients showed a substantial volumetric response. The response rate by body weight was comparable between the subgroup weighing less than 40 kg and that weighing 40 kg or more, supporting the appropriateness of the body surface area-based dosing strategy used in this study. In the pain score analysis restricted to patients with a baseline score of ≥10 mm (*n* = 12), scores tended to decrease after serabelisib treatment, and some patients showed improved performance status compared with baseline. By contrast, QOL scores did not improve to a clinically relevant extent, and disease severity in the cosmetic and functional domains did not change notably. Thus, the radiological response was not accompanied by consistent improvements in symptoms, function, or QOL, and the ≥20% volumetric threshold has not been validated as a measure of clinically meaningful improvement in these diseases. For an approximately spherical lesion, a 20% reduction in volume corresponds to a reduction of only approximately 7% in linear dimensions, whereas the 30% decrease in diameter used to define partial response in solid tumors corresponds to a volume reduction of approximately 66% [42]. The ≥20% criterion should therefore be regarded as an indicator of radiological activity rather than as evidence of clinical improvement. Consistent with this interpretation, radiological responders did not show consistently greater improvements in pain, QOL, or performance status than non-responders in a post hoc descriptive comparison (Table S6). The lack of consistent clinical improvement may reflect the difficulty of evaluating symptoms and QOL in all patients with vascular malformations, because these diseases produce multiple and complex symptoms that differ in type, severity, and lesion location in each individual patient. The relationship between radiological response and clinical benefit should be investigated in future studies using disease-specific outcome measures.

The AEs designated as safety risks in this phase 3 study were hyperglycemia, increased blood creatine phosphokinase, and elevated ALT and AST. Hyperglycemia occurred in one patient and was considered treatment-related; however, no notable changes were observed in blood glucose or hemoglobin A1c through week 52. PI3Kα plays a crucial role in the insulin signaling pathway that regulates blood glucose levels [43], and hyperglycemia was observed in the 300 mg once-daily dosing group in a phase 1 study of serabelisib [21]. By contrast, no hyperglycemia was observed in the previous phase 2 study of serabelisib [22], suggesting that the degree of PI3Kα inhibition required for the efficacy of serabelisib may be less than that required to alter blood glucose regulation in patients with VM, LM, and KTS. Hyperinsulinemia, the most frequent ADR (13.9%), was mostly grade 1 and did not require dose modification; it may reflect a compensatory increase in insulin secretion in response to on-target PI3Kα inhibition, which is consistent with the stable blood glucose and hemoglobin A1c levels observed through week 52. AST and ALT elevations were reported in a previous phase 2 study, with a severity of grade 3 or higher in some patients [22], and these AEs were designated as safety risks of interest in the present phase 3 study. AST and ALT increases, each observed in two patients, were considered treatment-related. Overall, 13 of the 15 hepatic ADR events resolved during the study (Table S5). These findings indicate that hepatic enzyme elevations require regular laboratory monitoring and may be manageable with treatment interruption, dose modification, or discontinuation when necessary. Because hepatic ADRs occurred between days 23 and 288 (median, 59 days), liver function should be monitored throughout treatment rather than only during the initial months, particularly in patients who require long-term therapy. Nevertheless, grade 3 or 4 ADRs occurred in 22.2% of patients, including two grade 4 hepatic events, and the sample size was insufficient to characterize uncommon but potentially serious adverse reactions, such as the fatal tubulointerstitial nephritis, for which a causal relationship could not be completely excluded. In addition, patients with diabetes mellitus or hepatic or renal impairment were excluded, so the safety findings may not apply to these populations. In particular, because hyperinsulinemia was the most frequent ADR, the absence of clinically relevant hyperglycemia in this study should not be generalized to patients with diabetes mellitus or impaired glucose tolerance in whom PI3Kα inhibition may lead to hyperglycemia [43]; blood glucose and hemoglobin A1c should be monitored during prolonged treatment. Tubulointerstitial nephritis has not been described as an on-target effect of PI3Kα inhibition, but because drug-induced tubulointerstitial nephritis is often idiosyncratic, renal function should also be monitored, and this event should be specifically evaluated in future studies and via post-marketing surveillance. The long-term safety of serabelisib, particularly in pediatric patients who may require prolonged treatment, remains to be established.

Sirolimus, an mTOR inhibitor, is effective in the treatment of slow-flow vascular malformations and is used clinically; however, it also raises safety concerns, such as interstitial pneumonia and infection, owing to its immunosuppressive effects [10,11,12]. mTOR acts downstream in the PI3K/AKT/mTOR pathway and plays a crucial role in immune signaling [10]. PI3K has several isoforms. PI3Kα is ubiquitously expressed and is primarily involved in signaling by growth factors such as insulin, playing a central role in cell proliferation and glucose metabolism; PI3Kβ is ubiquitously expressed and is involved in platelet activation, thrombus formation, and proliferation in specific tumors; and PI3Kγ and PI3Kδ are primarily expressed in immune cells and regulate immune responses, inflammation, and cell migration [43]. Because it selectively inhibits the PI3Kα isoform, serabelisib could theoretically reduce immune-related off-target effects; however, this hypothesis requires confirmation in comparative studies.

For context, sirolimus has been evaluated in several prospective studies of slow-flow vascular malformations, although differences in populations, endpoints, and response definitions preclude direct comparison with the present study. In a phase 2 trial of complicated vascular anomalies, 47 of 57 evaluable patients had a partial response after 6 months, as determined by a composite of functional impairment, QOL, and radiological assessment, and grade 3 or higher sirolimus-related blood or bone marrow toxicity occurred in 27% of patients [44]. In a prospective phase 2 study of extensive slow-flow malformations, all 16 evaluable patients showed improvement in symptoms and QOL after 12 months, and mucositis was the most common grade 3 AE (10%) [45]. In the PERFORMUS trial of 59 children, changes in malformation volume on MRI did not differ overall between the sirolimus and observation periods, except for a significant decrease in pure LM, whereas pain, bleeding, oozing, and QOL improved, and oral ulcers occurred in 49.2% of patients [12]. In the present study, response was defined solely by volumetric reduction, QOL did not improve consistently, and oral ulceration was not among the frequent ADRs. Because there was no concurrent comparator, no conclusions can be drawn regarding the relative efficacy or safety of serabelisib and sirolimus, and comparative studies are needed.

The PK analyses demonstrated stable trough plasma concentrations of serabelisib from week 12 through week 52. This finding indicates that consistent systemic exposure can be achieved across a wide age range, including pediatric patients, which is essential for the long-term treatment of congenital vascular malformations.

This study has several limitations. The single-arm design with an external natural history comparator, rather than a concurrent control group, limits definitive conclusions regarding efficacy. The sample size was small, particularly for subgroup analyses by disease type, severity, and age, because of the rarity of these diseases. Moreover, differences in patient background between this study and the historical control cohort, such as symptom severity and previous treatment history, which were not comprehensively collected in this study, may limit comparability. In addition, genetic analyses were not performed, precluding evaluation of genotype–response relationships. Somatic PIK3CA variants are found in most LMs and in KTS, which belongs to the PIK3CA-related overgrowth spectrum, whereas approximately half of VMs are caused by TEK variants and approximately one-quarter by PIK3CA variants [13,14,15,16]. Because patients were selected on clinical grounds, the study population probably included patients with different causative variants; patients without PI3Kα pathway activation may have been less likely to respond, which could have diluted the overall response rate. Conversely, because TEK variants are also thought to activate PI3K/AKT signaling [13,14], a response to PI3Kα inhibition cannot be excluded in TEK-associated VM. The numerically different response rates among VM, LM, and KTS should therefore not be interpreted as evidence of disease- or genotype-specific efficacy, and molecular characterization of lesional tissue should be incorporated into future studies to guide patient selection. Furthermore, the study population was limited to Japanese patients, which may restrict the generalizability of the findings, and treatment was open-label, although target lesion volumes were assessed by a blinded independent central committee. The maximum treatment duration was 52 weeks; therefore, the long-term efficacy and safety of serabelisib remain to be established. Despite these limitations, the results of this study indicate that selective inhibition of PI3Kα with serabelisib has radiological activity in patients with VM, LM, and KTS; however, radiological activity should be distinguished from demonstrated clinical benefit, which, together with long-term safety, remains to be confirmed. Accordingly, the potential role of serabelisib in clinical practice, particularly in pediatric patients who may require long-term treatment, should be considered in light of the uncertainty of the historical comparison, the lack of consistent improvement in patient-reported outcomes, and the observed hepatic adverse reactions. Further accumulation of data is warranted to optimize patient selection, treatment duration, and long-term management strategies in real-world clinical practice.

## 5. Conclusions

In this phase 3 single-arm trial, serabelisib administered once daily for at least 24 weeks and up to 52 weeks produced a response rate in target lesion volume that significantly exceeded a prespecified natural history-based null hypothesis rate in patients with VM, LM, and KTS. This finding indicates radiological activity; however, improvements in symptoms and QOL were not consistently observed, and clinical benefit has not been demonstrated. Hepatic ADRs, including grade 3 or 4 events, were the principal risk and mostly resolved with treatment interruption or discontinuation, indicating the need for regular laboratory monitoring. Larger controlled studies with longer follow-up and molecular characterization are warranted to confirm the clinical benefit and long-term safety of serabelisib, particularly in pediatric patients.

## Figures and Tables

**Table 1:** Patient characteristics.

Characteristics			Statistics *n* (%)	
N			36	
Sex	Male		20	(55.6)
	Female		16	(44.4)
Age, years	Mean ± SD		21.6 ± 18.0	
	Median (min–max)		15.5 (2–62)	
	Age group			
	By adult/minor	<18	20	(55.6)
		≥18	16	(44.4)
	By pediatric age	<12	13	(36.1)
		≥12	23	(63.9)
Weight, kg	Mean ± SD		44.4 ± 23.0	
	Median (min–max)		46.7 (11.1–93.9)	
BMI, kg/m^2^	Mean ± SD		19.7 ± 3.9	
	Median (min–max)		19.2 (13.6–33.5)	
Disease type	VM		21	(58.3)
	LM		3	(8.3)
	KTS		12	(33.3)
Non-target lesion	Presence		10	(27.8)
	Absence		26	(72.2)

Analyzed in the full analysis set. KTS, Klippel–Trenaunay syndrome; LM, lymphatic malformation; max, maximum; min, minimum; SD, standard deviation; VM, venous malformation.

**Table 2:** Overview of adverse event incidence.

AE Incidence, *n* (%)	AEs		ADRs	
N	36		36	
Overall	36	(100.0)	19	(52.8)
Serious	6	(16.7)	0	
Grade 1	1	(2.8)	5	(13.9)
Grade 2	20	(55.6)	6	(16.7)
Grade 3	12	(33.3)	6	(16.7)
Grade 4	2	(5.6)	2	(5.6)
Grade 5	1	(2.8)	0	
AEs with an incidence of ≥10% (PT)				
Nasopharyngitis	24	(66.7)	0	
Influenza	6	(16.7)	0	
Pyrexia	6	(16.7)	2	(5.6)
Gastroenteritis	5	(13.9)	0	
COVID-19	5	(13.9)	0	
Hyperinsulinemia	5	(13.9)	5	(13.9)
Nausea	5	(13.9)	4	(11.1)
Liver function test abnormality	4	(11.1)	4	(11.1)

Analyzed in the full analysis set. Coded according to the Medical Dictionary for Regulatory Activities version 28.0. ADR, adverse drug reaction; AE, adverse event; PT, preferred term.

## Data Availability

The original contributions presented in this study are included in the article. Further inquiries can be directed to the corresponding author.

## Supplementary Materials

The following supporting information can be downloaded at https://www.mdpi.com/article/10.3390/jcm15218624/s1: Figure S1: Changes in the target lesion volume from baseline in each patient at the final evaluation point; Figure S2: Changes in the plasma trough concentrations of serabelisib through 52 weeks; Table S1: Changes in the QOL scores through 52 weeks; Table S2: Changes in blood glucose and hemoglobin A1c through 52 weeks; Figure S3: The volumetric measurement procedure for the target lesion; Table S3: Post hoc sensitivity analyses of the primary endpoint; Table S4: Baseline lesion characteristics and absolute target lesion volumes; Table S5: Details of hepatic adverse drug reactions; Figure S4: Bland–Altman plots of interobserver agreement in target lesion volume; Table S6: Changes in pain scores, QOL scores, and performance status by response status.
