# Supplementary material for: Serabelisib, a Selective PI3Kα Inhibitor, for Slow-Flow Vascular Malformations in a Phase 3 Multicenter Single-Arm Study with Natural History Comparison
Source: J Clin Med. 2026 Nov 5;15(21):8624. doi: 10.3390/jcm15218624 (PMC13763688; doi:10.3390/jcm15218624)
Supplement: Supplementary file 1 [file jcm-15-08624-s001.zip › jcm-4657109-supplementary.pdf]

Supplementary Materials

Serabelisib, a Selective PI3Kα Inhibitor, for Slow-Flow Vascular Malformations in a Phase 3 Multicenter Single-Arm Study with Natural History Comparison

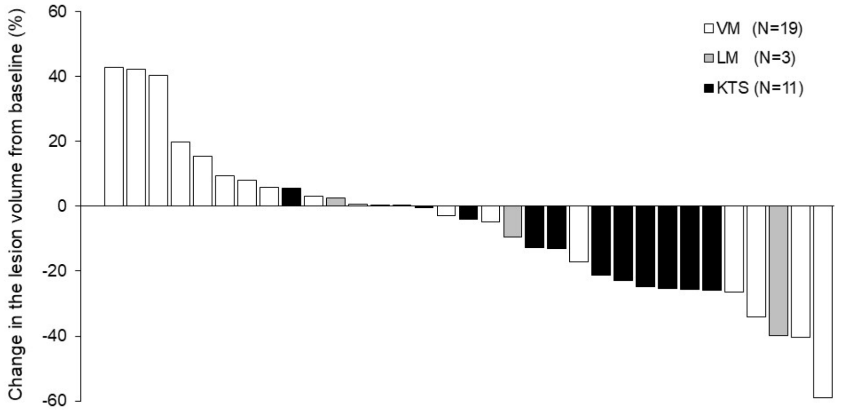

Figure S1. Changes in the target lesion volume from baseline in each patient at the final evaluation point.

The change in each patient with venous malformation (VM), lymphatic malformation (LM), and Klippel–Trenaunay syndrome (KTS) is shown using a waterfall plot.

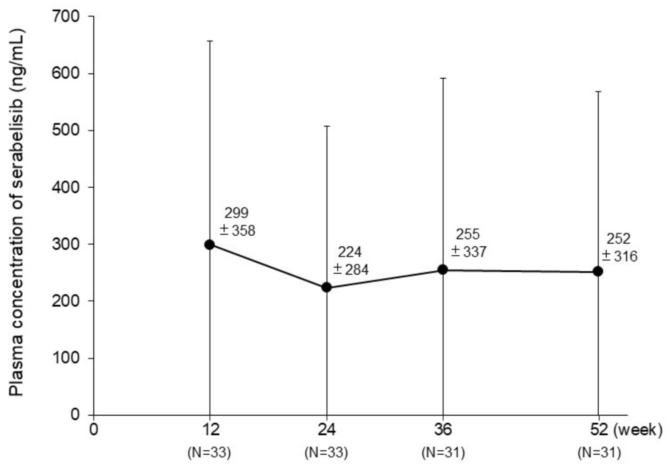

Figure S2. Changes in the plasma trough concentrations of serabelisib through 52 weeks.

Data are expressed as mean ± standard deviation.

Table S1. Changes in the QOL scores through 52 weeks.

QOL scores		Baseline	Week 52	Change
PedsQL	Parent proxy report (2–18 years old)	82.17 ± 18.18 (19)	84.88 ± 19.74 (19)	3.41 ± 9.94 (18)
	Patient report (5–25 years old)	85.80 ± 16.30 (16)	88.86 ± 15.41 (16)	3.55 ± 7.41 (15)
SF-36v2	Patient report (≥26 years old)			
	Physical	47.02 ± 9.87 (11)	45.80 ± 13.00 (8)	0.30 ± 12.00 (7)
	Mental	48.46 ± 12.07 (11)	44.29 ± 8.47 (8)	−3.41 ± 6.37 (7)
	Role/social	49.54 ± 8.57 (11)	43.09 ± 11.19 (8)	−7.21 ± 8.51 (7)
EQ-5D-Y	Parent proxy report (4–7 years old)	0.80 ± 0.09 (4)	0.91 ± 0.09 (5)	0.11 ± 0.12 (4)
	Patient report (8–15 years old)	0.90 ± 0.16 (8)	0.91 ± 0.21 (7)	0.01 ± 0.05 (7)
EQ-5D-5L	Patient report (≥16 years old)	0.86 ± 0.14 (17)	0.77 ± 0.21 (14)	−0.10 ± 0.15 (13)

Data are expressed as mean ± standard deviation with the evaluated number of patients (or their parents).

EQ-5D, EuroQol 5-Dimension; PedsQL, Pediatric Quality of Life Inventory; QOL, quality of life; SF-36v2, 36-Item Short Form Health Survey version 2.

Table S2. Changes in blood glucose and hemoglobin A1c through 52 weeks.

	**Blood glucose (mmol/L)**	**Hemoglobin A1c (fraction of 1)**
Baseline	5.15 ± 0.43 (36)	0.0534 ± 0.0030 (36)
Week 12	5.07 ± 0.50 (35)	0.0537 ± 0.0031 (35)
Week 24	5.09 ± 0.34 (34)	0.0535 ± 0.0031 (34)
Week 36	5.11 ± 0.50 (31)	0.0534 ± 0.0029 (31)
Week 52	5.06 ± 0.43 (31)	0.0536 ± 0.0030 (31)

Data are expressed as mean ± standard deviation with the evaluated number of patients. The blood glucose was measured at least 2 hours after eating.

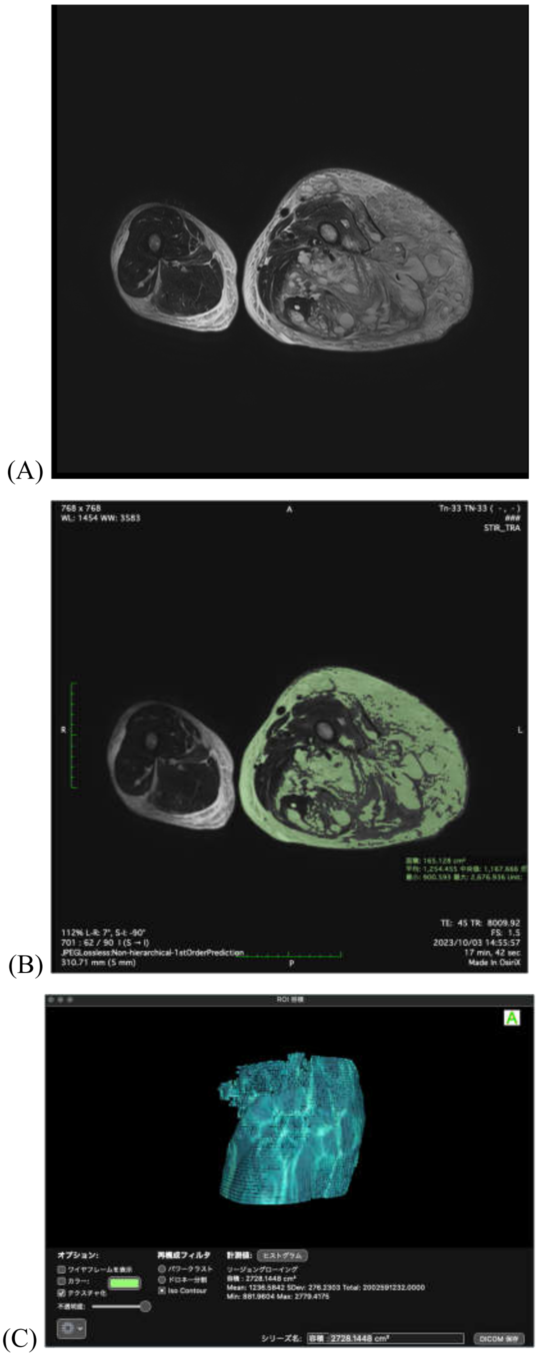

Figure S3. Volumetric measurement procedure for the target lesion.

(A) Standardized MRI acquisition at baseline: A representative axial short tau inversion recovery (STIR) image. MRI was performed using a standardized protocol, ensuring that each patient was scanned with the same device and identical parameters across all time points. Images were acquired with a slice thickness of 3–5 mm (up to 8 mm for large lesions) without an interslice gap.

(B) Lesion segmentation and manual refinement: Experienced radiologist reviewers, blinded to clinical information, consulted with the Chair of the Image Evaluation Committee to define the measurement range based on bony and anatomical landmarks. The lesion contour (ROI) was captured on each slice using the region-growing tool of OsiriX MD software. The manual brush and pencil tools were used to refine the contour and exclude non-lesional components (e.g., bone or large vessels), ensuring an accurate assessment of the infiltrative boundary.

(C) Final volumetric calculation and 3D rendering: Three-dimensional reconstruction of the segmented lesion. The total volume was calculated as the sum of the delineated areas multiplied by the slice thickness.

Table S3. Post hoc sensitivity analyses of the primary endpoint.

Analysis	Comparator / assumption	Responders, n/N (%)	Comparator value	p-Value
Primary analysis (prespecified)	Null hypothesis rate	11/36 (30.6)	6.7%	<0.001 [a]
Alternative null hypothesis rate	LM survey benchmark [27]	11/36 (30.6)	1.7%	<0.001 [b]
	Alternative rate	11/36 (30.6)	10%	<0.001 [b]
	Alternative rate	11/36 (30.6)	15%	0.014 [b]
	Alternative rate	11/36 (30.6)	20%	0.089 [b]
	Upper 95% confidence limit of the natural history rate	11/36 (30.6)	22.1%	0.15 [b]
Tipping point	Highest null rate with one-sided $p < 0.025$	11/36 (30.6)	16%	0.022 [b]
Comparison with the natural history cohort	All patients vs. natural history cohort	11/36 (30.6)	2/30 (6.7)	0.027 [c]
	VM and KTS only vs. natural history cohort	10/33 (30.3)	2/30 (6.7)	0.024 [c]
Bayesian analysis	Posterior probability that the response rate with serabelisib exceeds the natural history rate (Jeffreys priors)	11/36 (30.6)	2/30 (6.7)	Probability 0.994 [d]
Completers	Patients who completed 52 weeks	11/31 (35.5; 95% CI, 19.2–54.6)	6.7%	<0.001 [a]
Week 24 (prespecified secondary endpoint)	All patients	8/36 (22.2; 95% CI, 10.1–39.2)	6.7%	0.004 [a]

[a] Two-sided exact binomial test. [b] One-sided exact binomial test. [c] Two-sided Fisher's exact test; the natural history cohort included patients with VM or KTS [22,23]. [d] Beta(0.5, 0.5) priors for both proportions; median difference in response rates, 23.0% (95% credible interval, 5.5%–40.6%). All sensitivity analyses were performed post hoc. CI, confidence interval; KTS, Klippel–Trenaunay syndrome; LM, lymphatic malformation; VM, venous malformation.

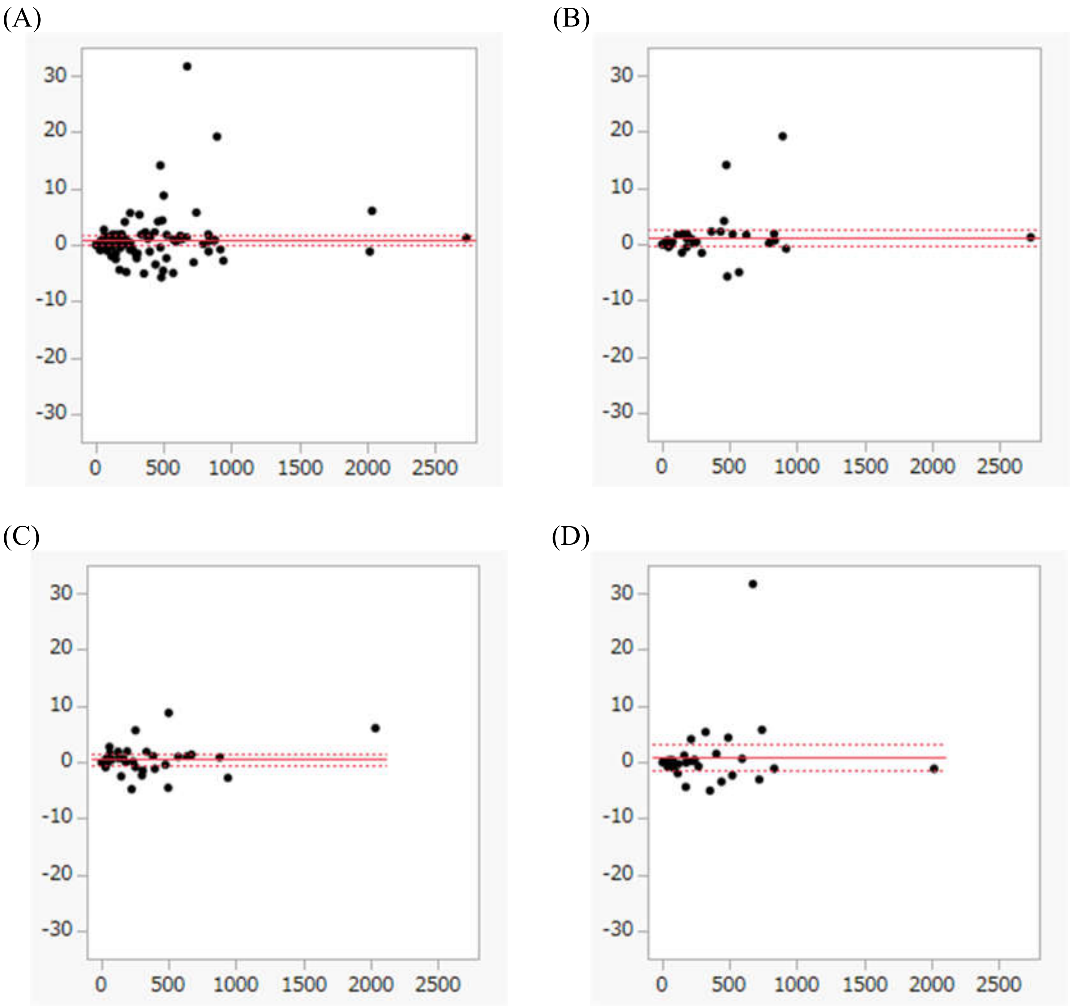

Figure S4. Bland–Altman plots of interobserver agreement in target lesion volume.

The x-axis shows the mean of the two reviewers' measurements, and the y-axis shows the difference between their measurements (Reviewer 1 minus Reviewer 2), in cm^3. Each point represents one paired measurement. The solid horizontal line indicates the mean difference (bias), and the dotted horizontal lines indicate the 95% confidence interval (CI) of the bias; the 95% limits of agreement (LoA) are given below. (A) Pooled analysis of all 101 paired measurements across visits: bias, 0.90 cm^3 (95% CI, 0.01 to 1.79); 95% LoA, −7.93 to 9.74 cm^3. (B) Screening ($n = 36$): bias, 1.24 cm^3 (95% CI, −0.19 to 2.68); 95% LoA, −7.07 to 9.55 cm^3. (C) Week 24 ($n = 34$): bias, 0.49 cm^3 (95% CI, −0.44 to 1.42); 95% LoA, −4.73 to 5.71 cm^3. (D) Week 52 ($n = 31$): bias, 0.96 cm^3 (95% CI, −1.32 to 3.24); 95% LoA, −11.22 to 13.15 cm^3. Panel A pools measurements across visits and does not adjust for within-patient correlation. In the visit-specific analyses of percentage differences (difference divided by the mean of the two measurements), the bias was 0.33% (95% LoA, −1.38% to 2.04%) at screening, 0.24% (95% LoA, −2.49% to 2.97%) at week 24, and 0.01% (95% LoA, −2.53% to 2.54%) at week 52.

Table S4. Baseline lesion characteristics and absolute target lesion volumes.

Characteristic	All (N = 36)	VM (n = 21)	LM (n = 3)	KTS (n = 12)
Target lesion location, % (n/N)				
Lower limb	33.3% (12/36)	23.8% (5/21)	0.0% (0/3)	58.3% (7/12)
Upper limb	22.2% (8/36)	14.3% (3/21)	0.0% (0/3)	41.7% (5/12)
Head and neck	33.3% (12/36)	47.6% (10/21)	66.7% (2/3)	0.0% (0/12)
Trunk	11.1% (4/36)	14.3% (3/21)	33.3% (1/3)	0.0% (0/12)
Disease severity at baseline, median (range)				
Cosmetic domain	3 (1–5)	2 (1–5)	4 (2–5)	4 (2–5)
Functional domain	3 (0–5)	3 (0–5)	3 (1–3)	3 (0–5)
Target lesion volume, cm^3, median (range)				
Baseline	241.2 (3.1–2728.8)	169.3 (3.1–895.0)	295.9 (78.3–919.6)	492.5 (149.2–2728.8)
Week 24	227.2 (4.1–2035.9)	129.1 (4.1–941.7)	303.4 (62.6–880.0)	361.9 (149.4–2035.9)
Week 52	216.5 (4.4–2019.7)	122.0 (4.4–742.2)	440.2 (47.1–833.2)	363.9 (117.6–2019.7)
Response rate by baseline volume, % (n/N)				
<200 cm3 (n = 15)	26.7% (4/15)	7.7% (1/13)	100.0% (1/1)	100.0% (1/1)
200 to <1000 cm3 (n = 19)	31.6% (6/19)	28.6% (2/7)	0.0% (0/2)	40.0% (4/10)
≥1000 cm3 (n = 1)	100.0% (1/1)	-	-	100.0% (1/1)

Analyzed in the full analysis set. KTS, Klippel–Trenaunay syndrome; LM, lymphatic malformation; VM, venous malformation.

Disease severity at baseline is shown as the median (range) of the maximum severity score across all sub-items within each domain for each patient. Target lesion volumes are shown as median (range); the baseline volume was not evaluable in one patient with VM.

Table S5. Details of hepatic adverse drug reactions.

Patient	Age/sex	ADR (PT)	Max grade	Onset (day)	Action taken	Days to resolution	Outcome
1	28/M	Drug-induced liver injury	4	23	Drug interrupted	35	Resolved
1	28/M	Liver function test abnormal	3	92	Drug withdrawn	15	Resolved
2	46/M	Liver function test increased	4	54	Drug interrupted	66	Resolved
2	46/M	Liver function test increased	3	145	Drug interrupted	-	Not resolved
3	38/M	Hepatic enzyme increased	3	59	Dose reduced	132	Resolved
4	51/F	Liver function test abnormal	3	29	Drug interrupted	57	Resolved
5	36/F	Liver function test increased	3	32	Drug interrupted	78	Resolved
5	36/F	Liver function test increased	3	158	Dose reduced	271	Resolved
6	35/F	Liver function test abnormal	2	85	Drug interrupted	92	Resolved
7	53/M	Liver function test increased	2	56	Dose reduced	57	Resolved
8	38/M	ALT increased	2	57	Dose reduced	253	Resolved
8	38/M	AST increased	1	85	Dose reduced	169	Resolved
9	2/M	ALT increased	2	288	Dose reduced	29	Resolved
9	2/M	AST increased	1	288	Dose reduced	29	Resolved
10	49/F	Liver function test abnormal	1	50	Dose not changed	-	Unknown

ADR, adverse drug reaction; ALT, alanine aminotransferase; AST, aspartate aminotransferase; PT, preferred term.

Patient 2: The second event of liver function test increased was classified as “not resolved” because some laboratory values remained slightly above the reference range; however, the event improved sufficiently to allow resumption of the study drug.

Patient 10: The outcome was classified as “unknown” because the patient died of an event judged by the investigator to be unrelated to the study drug (tubulointerstitial nephritis), which precluded further follow-up of this adverse drug reaction.

Table S6. Changes in pain scores, QOL scores, and performance status by response status.

		Baseline	Week 52	Change
Pain score (VAS, mm)	Responder	17.63 ± 26.47 (8)	25.88 ± 35.58 (8)	8.25 ± 22.86 (8)
	Non-responder	18.95 ± 24.43 (19)	15.87 ± 26.25 (15)	1.64 ± 17.76 (14)
PedsQL	Parent proxy report (2–18 years old)			
	Responder	76.20 ± 29.02 (5)	81.54 ± 29.80 (5)	5.34 ± 10.83 (5)
	Non-responder	84.30 ± 13.41 (14)	86.07 ± 16.14 (14)	2.66 ± 9.94 (13)
	Patient report (5–25 years old)			
	Responder	80.98 ± 20.71 (4)	78.53 ± 24.21 (4)	−2.45 ± 4.97 (4)
	Non-responder	87.41 ± 15.29 (12)	92.30 ± 10.58 (12)	5.73 ± 7.06 (11)
SF-36v2	Responder			
	Physical	45.60 ± 11.41 (4)	46.93 ± 13.73 (4)	1.33 ± 7.29 (4)
	Mental	51.35 ± 11.11 (4)	47.15 ± 5.76 (4)	−4.20 ± 6.51 (4)
	Role/social	45.93 ± 9.36 (4)	41.70 ± 9.84 (4)	−4.23 ± 9.7 (4)
	Non-responder			
	Physical	47.83 ± 9.76 (7)	44.68 ± 14.22 (4)	−1.07 ± 18.63 (3)
	Mental	46.81 ± 13.13 (7)	41.43 ± 10.60 (4)	−2.37 ± 7.43 (3)
	Role/social	51.60 ± 8.07 (7)	44.48 ± 13.79 (4)	−11.20 ± 5.86 (3)
EQ-5D-5L utility score	Responder	0.90 ± 0.12 (6)	0.78 ± 0.27 (6)	−0.12 ± 0.18 (6)
	Non-responder	0.84 ± 0.15 (11)	0.76 ± 0.16 (8)	−0.09 ± 0.14 (7)
EQ-5D-Y utility score	Responder	0.76 ± 0.35 (2)	0.72 ± 0.39 (2)	−0.03 ± 0.04 (2)
	Non-responder	0.95 ± 0.04 (6)	0.99 ± 0.03 (5)	0.03 ± 0.04 (5)
Lansky play-performance scale	Responder (n = 5)			Improved: 3<br>No change: 1<br>Worsening: 1
	Non-responder (n = 13)			Improved: 4<br>No change: 7<br>Worsening: 2
Karnofsky performance scale	Responder (n = 6)			Improved: 2<br>No change: 4<br>Worsening: 0
	Non-responder (n = 7)			Improved: 2<br>No change: 5<br>Worsening: 0

Data are expressed as mean ± standard deviation (number of evaluable patients). Responders were defined as patients with a ≥20% reduction from baseline in target lesion volume at the final evaluation point (n = 11); all other patients were classified as non-responders (n = 25). Higher scores indicate more severe pain for the VAS and better health status for the other instruments. For the SF-36v2, Physical, Mental, and Role/social denote the corresponding component summary scores. All analyses were performed post hoc. EQ-5D, EuroQol 5-Dimension; PedsQL, Pediatric Quality of Life Inventory; QOL, quality of life; SF-36v2, 36-Item Short Form Health Survey version 2; VAS, visual analog scale.
For the Lansky play-performance scale (younger than 17 years) and the Karnofsky performance scale (17 years and older), the numbers of patients whose scores improved, did not change, or worsened from baseline to week 52 are shown.
